# Rectal cancer in the era of precision oncology: more options, more uncertainty

**DOI:** 10.1093/bjs/znag059

**Published:** 2026-05-11

**Authors:** Reza Mirnezami

**Affiliations:** Department of Colorectal Surgery, Royal Free Hospital, London, UK

Rectal cancer management is entering a period of profound change. Advances in systemic therapy, radiotherapy, and surgical technique have expanded the therapeutic arsenal, transforming what was once a relatively linear treatment pathway into a far more complex clinical decision framework. In many cases, the challenge is no longer determining whether treatment is required but deciding which of several competing strategies should be pursued.

Consider a patient with a mid-rectal cancer staged on MRI as T3a N1 with a clear predicted circumferential resection margin (CRM) and no extramural vascular invasion (EMVI)—a tumour that sits squarely within the ‘grey zone’ between clearly early and clearly locally advanced disease. The tumour is technically resectable with total mesorectal excision (TME). According to contemporary international guidance, including the latest National Comprehensive Cancer Network (NCCN) and European Society For Medical Oncology (ESMO) rectal cancer guidelines, this patient could reasonably be offered immediate surgery with selective postoperative therapy, neoadjuvant chemoradiotherapy followed by surgery, or total neoadjuvant therapy (TNT) before resection^1,2^. In principle, such flexibility reflects welcome progress towards personalized rectal cancer care. In practice, however, it introduces a new challenge. When several treatment strategies are all acceptable, deciding which path to follow becomes far less straightforward^3-6^. Although guidelines emphasize that multiple options should be discussed with the patient, the way in which options are framed is rarely neutral. Treatment recommendations are inevitably influenced by clinician experience, institutional practice patterns, and local expertise. In addition, although major guideline bodies broadly agree on the principles of modern rectal cancer management, subtle differences in conceptual framing can shape clinical decision-making. For example, NCCN guidance often presents several parallel treatment pathways as acceptable options for stage II–III rectal cancer, whereas ESMO guidelines tend to emphasize risk stratification and treatment sequencing^1,2^. These distinctions may appear modest on paper but can influence how options are prioritized within multidisciplinary discussions.

The dilemma illustrated by this clinical vignette is not new, and a useful way to understand it is by revisiting a familiar clinical shorthand: the distinction between ‘good tumours’, ‘bad tumours’, and the grey zone in between. Historically, this informal framework provided a pragmatic guide for treatment planning. Early tumours (the ‘good’) were managed with local excision or primary surgery. At the opposite extreme, clearly locally advanced cancers threatening the CRM (the ‘bad’) would receive multimodality therapy before definitive resection. Between these poles lay intermediate disease, where treatment was once more predictable. Today, however, that middle ground has become far less certain, and it is within this intermediate space that recent therapeutic developments have begun to reshape decision-making. The increasing adoption of TNT has expanded the role of preoperative treatment and randomized trials such as RAPIDO and PRODIGE-23 have demonstrated improved oncological outcomes when chemotherapy is incorporated into the preoperative phase^7,8^. More recently, attention has also focused on how treatment sequencing within TNT may influence the likelihood of organ preservation^5^. Historically, complete clinical responses were largely opportunistic, occurring in patients who received neoadjuvant therapy for oncological indications such as threatened margins or clearly locally advanced disease. The emergence of TNT, however, has begun to shift this dynamic. By delivering systemic chemotherapy before resection and increasing tumour response rates, TNT has made organ preservation a more deliberate therapeutic objective. As a result, multidisciplinary teams are increasingly considering intensified neoadjuvant strategies even for patients with technically operable rectal cancers who might previously have proceeded directly to surgery.

This evolution reflects a growing recognition of the long-term functional consequences of rectal cancer surgery. Although TME is undeniably oncologically effective, it is associated with substantial morbidity, including low anterior resection syndrome, sexual dysfunction and, in some cases, permanent stoma formation. For patients with distal tumours in particular, these outcomes can have a profound impact on quality of life. It is therefore unsurprising that both clinicians and patients are increasingly attracted to strategies that may allow organ preservation where oncologically safe.

The most widely adopted of these strategies is non-operative management following a complete clinical response, commonly referred to as the watch-and-wait approach. Data from large international registries and prospective studies suggest that carefully selected patients achieving a complete clinical response after neoadjuvant therapy can be managed safely with structured surveillance, with most local regrowths occurring early and remaining amenable to salvage surgery^9,10^. The potential of TNT to increase complete response rates and facilitate organ preservation is therefore a focus of contemporary discussion^11^. For many patients, this approach offers the possibility of avoiding radical pelvic surgery without compromising long-term oncological outcomes. Yet the increasing use of neoadjuvant treatment with the intention of achieving organ preservation raises an important issue. Although some patients may ultimately avoid major surgery, others will undergo intensified treatment only to proceed to resection regardless. In these cases, patients are exposed to the potential toxicity of both systemic therapy and surgery. Determining when such treatment escalation is justified therefore represents one of the central challenges of contemporary rectal cancer care.

A second and equally difficult question arises in patients who achieve a complete clinical response despite presenting with features traditionally associated with high-risk disease. Tumours characterized by EMVI, nodal involvement, or threatened margins have historically been regarded as poor candidates for organ preservation. However, with the increasing use of TNT, complete radiological and clinical responses are now observed even in tumours that initially appeared biologically aggressive. For multidisciplinary teams, this creates a new and complex challenge. Should the decision to proceed with surgery be guided primarily by the biological risk suggested by initial staging, or by the degree of response achieved following treatment? A patient presenting with T3 N2 disease with EMVI would typically be considered a clear candidate for multimodality therapy followed by radical resection. Yet if that patient subsequently demonstrates a complete clinical response after TNT, the oncological rationale for proceeding to resection becomes less certain.

At present, evidence to guide these decisions is limited. Although observational data support the safety of watch-and-wait in carefully selected patients, most series include heterogeneous populations and relatively few patients with initially high-risk disease. As a result, practice varies considerably between centres. Some multidisciplinary teams remain cautious, prioritizing initial tumour biology when considering organ preservation. Others place greater emphasis on treatment response, particularly when surveillance and salvage surgery can be delivered within experienced centres.

Taken together, these developments signal a shift in rectal cancer management, away from rigid treatment algorithms and towards a more nuanced model of decision-making shaped by clinical judgement and patient preference. Advances in systemic therapy, radiotherapy, and organ-preserving strategies have expanded the therapeutic landscape, creating new opportunities for individualized care while simultaneously introducing new uncertainties. Navigating this uncertainty will require careful multidisciplinary judgement, transparent discussions with patients and continued evaluation of outcomes within prospective studies and international registries. Ultimately, the success of modern rectal cancer care will depend not on the number of therapeutic options available, but on how wisely we use them.

## Previous presentation

This paper is not based on a previous communication to a society or meeting.

## References

[1] Benson AB, Venook AP, Adam M, Ambelil M, Bray A, Chang G et al NCCN Clinical Practice Guidelines in Oncology (NCCN Guidelines), Rectal Cancer Version 2.2026. 2026:1–166

[2] Hofheinz RD, Fokas E, Benhaim L, Price TJ, Arnold D, Beets-Tan R et al Localised rectal cancer: ESMO clinical practice guideline for diagnosis, treatment and follow-up. Ann Oncol 2025;36:1007–1024 40412553 10.1016/j.annonc.2025.05.528

[3] Harris DA . T3 N1 M0 rectal cancer: optimal initial management is upfront surgery. BJS 2024;111:znad321 37995255 10.1093/bjs/znad321

[4] Nilsson PJ . T3 N1 M0 rectal cancer: optimal initial management is total neoadjuvant therapy. BJS 2024;111:znad322 37995257 10.1093/bjs/znad322

[5] Arthur C, Teo M. T3 N1 M0 rectal cancer: the optimal initial management is long-course chemoradiotherapy. BJS 2024;111:znad323 37995256 10.1093/bjs/znad323

[6] Braun M . T3N1M0 rectal cancer: the optimal initial management is systemic anti-cancer therapy. BJS 2024;111:znad324 37995273 10.1093/bjs/znad324

[7] Bahadoer RR, Dijkstra EA, van Etten B, Marijnen CAM, Putter H, Kranenbarg EMK et al Short-course radiotherapy followed by chemotherapy before total mesorectal excision (RAPIDO): a randomised phase 3 trial. Lancet Oncol 2021;22:29–42 33301740 10.1016/S1470-2045(20)30555-6

[8] Conroy T, Bosset JF, Etienne PL, Rio E, François É, Mesgouez-Nebout N et al Neoadjuvant chemotherapy with FOLFIRINOX and chemoradiotherapy in locally advanced rectal cancer (PRODIGE-23). Lancet Oncol 2021;22:702–715 33862000 10.1016/S1470-2045(21)00079-6

[9] Garcia-Aguilar J, Patil S, Gollub MJ, Kim JK, Yuval JB, Thompson HM et al Organ preservation in patients with rectal adenocarcinoma treated with total neoadjuvant therapy (OPRA). J Clin Oncol 2022;40:2546–2556 35483010 10.1200/JCO.22.00032 PMC9362876

[10] van der Valk MJM, Hilling DE, Bastiaannet E, Meershoek-Klein Kranenbarg E, Beets GL, Figueiredo NL et al Long-term outcomes of clinical complete responders after neoadjuvant treatment for rectal cancer in the international watch & wait database. Lancet 2018;391:2537–2545 29976470 10.1016/S0140-6736(18)31078-X

[11] Grotenhuis BA, Couwenberg AM, Marijnen CAM. Total neoadjuvant therapy for organ preservation in rectal cancer. Ann Oncol 2024;35:343–345 10.1016/j.annonc.2024.09.014 39461746

